# Genetic polymorphism of *Pit-1* and *CSN3* genes in Holstein calves and its associations with calf birth weight

**DOI:** 10.5194/aab-65-285-2022

**Published:** 2022-08-04

**Authors:** Ismail Fındık, Memis Özdemir

**Affiliations:** Department of Animal Science, Faculty of Agriculture, Atatürk University, 25240 Erzurum, Türkiye

## Abstract

The aim of this study was to examine the polymorphic structures of *Pit-1* and *CSN3* genes of Holstein calves bred in Gümüşhane province of Türkiye, to determine the distribution of genotype and allele gene frequencies, as well as examine the effects of determined polymorphisms on birth weight of calves. *Hinf*I polymorphisms of *Pit-1* and *CSN3* genes were identified in DNA isolated from blood samples of 100 Holstein calves used in the study, using the PCR-RFLP method. According to the Hardy–Weinberg genetic equilibrium test, it was observed that the distribution of genotype frequencies of *Hinf*I polymorphisms of *Pit-1* genes in the studied population was in equilibrium, but not in equilibrium in terms of *CSN3* gene location. The AA, AB, and BB genotype frequencies of the *Pit-1* gene in the population were 13.4 %, 40.2 %, and 46.3 %, respectively; the frequency of the A allele was 0.34, while for B allele it was 0.66. The AA, AB, and BB genotype frequencies of the *CSN3* gene were found to be 24.5 %, 36.7 %, and 38.8 %, respectively; the frequency of the A allele was 0.43 and the frequency of the B allele was 0.57. According to the Hardy–Weinberg genetic equilibrium test, the distribution of genotype frequencies was in equilibrium in the *Pit-1*/*Hinf*I polymorphism, but not in the *CSN3*/*Hinf*I polymorphism. A statistically significant relationship was not found between the genotypes of both polymorphic regions and calf birth weight.

## 1 Introduction

Individuals with superior fertility and milk yield are very important for the profitability of cattle enterprises. Economic yield traits are quantitative traits with low heritability and polygenic inheritance. For this reason, it is very long and costly to achieve the targeted genetic improvement with classical selection methods in such traits. The disadvantages of classical selection methods in the improvement of such quantitative traits can only be eliminated by using molecular markers (Erhardt and Weimann, 2007; Özdemir and Doğru, 2008). It is thought that the success rate may increase in improving some important yield characteristics of animals, such as health and welfare, by utilizing some genes, which are called “marker genes” in farm animals and whose relationship with the investigated phenotypes is used for breeding purposes. By using genetic markers in selection, it can accelerate the selection process, increase the quality of agricultural production, reduce the production cost, and compete with other manufacturers.

The SNPs of candidate genes as a marker may exert their effect on associated traits through changing of gene expression. The use of genetic polymorphisms and molecular markers has been reported to significantly increase the speed and efficiency of livestock selection and breeding (Dario et al., 2009; Zhang et al., 2013). Candidate genes are generally selected because of their physiological or biological effects on quantitative traits such as milk yield and body weight gain or their physical association with genes that influence these traits (Unanian et al., 2002). Many of the characteristics that affect animal productivity are complex and greatly influenced by environmental factors, such as the feeding and care of animals. However, recent developments in molecular biology and biotechnology indicate that marker-assisted selection (MAS) will provide more accurate and effective selection of yield traits (Hua et al., 2009; Litwinczuk and Krol, 2002).

Genetic markers are evaluated in two ways as direct gene markers and linked gene markers. While direct gene markers are defined as variants in the coding or non-coding DNA sequence within a gene region, linked gene markers are defined as the coexistence of one or more genes on the same arm of the chromosome (Hetzel, 2004). Genetic markers that can be used in breeding programs based on selection by increasing the frequency of desired genes in the population are particularly useful for breeding quantitative traits that are expensive and difficult to measure accurately or that can be seen later in life or only in one sex.

The *CSN3* ( κ -casein) gene has a crucial role in milk quality and coagulation, as well as in the formation, stabilization, and aggregation of casein micelles. Therefore, genetic variants of *CSN3* are associated with protein content of milk, cheese yield, and yield frequency and have a significant effect on coagulation time. The great impact of *CSN3* on milk production has led to numerous studies on this gene region such as cattle, goats, sheep, and buffalo (Othman et al., 2011; Feligini et al., 2005; Ren et al., 2011). Bovine *CSN3* is located on chromosome 6 (6q31) and has a total length of 13 kb. It contains 5 exons and 4 introns, and most of the mature protein coding sequences are in exon 4 (Khaizaran and Al-Razem, 2014). The most common A and B alleles of *CSN3*, which has many variants, are found and studied. Codons 136 and 148 of *CSN3*, consisting of 169 amino acids, are determinants for this allelic variation. As a matter of fact, while there is threonine (ACC) at codon 136 in A allele and aspartic acid (GAT) at codon 148, there is isoleucine (ATC) and alanine (GTC) in B allele, respectively (Kaminski, 1996). This allelic difference can be easily detected by some restriction endonucleases (*Alu*I, *Hind*III, *Hinf*I, *Taq*I) (Doğru et al., 2008).

In many studies on the polymorphic structure of the *CSN3* gene, it has been reported that the frequency of the A allele gene is generally higher in many breeds (Lunden et al., 1997; Strzalkowska et al., 2002; Özdemir and Doğru, 2005; Caroli et al., 2009). In studies in which *CSN3* polymorphism is associated with yield traits, it has been reported that cattle with *CSN3* B allele have higher milk yield, protein yield, fat yield, and fat percentage than cattle with A allele, and that other milks give better results than cheese production (Hu and Mao, 1995; Özdemir and Doğru, 2005); it has been suggested that by increasing the frequency of the *CSN3* B gene, significant progress can be made in the improvement of milk yield traits in cattle (Lunden et al., 1997; Strzalkowska et al., 2002; Özdemir and Doğru, 2005; Caroli et al., 2009).

Pituitary specific transcription factor-1 (pituitary specific transcription factor-1, *Pit-1* or *POU1F1*), which is on the first chromosome in cattle, weighs approximately 33 kDa, has 5 introns and 6 exons, and consists of 291 amino acids. It is a pituitary-specific transcription factor responsible for its secretion (Renaville et al., 1997). *Pit-1* plays a role in pituitary development and proliferation of somatic cells and secretion of growth hormone (GH) and prolactin (PRL) hormones in mammals (Zhang et al., 2009; Aytekin and Boztepe, 2013). Absence or lower expression of *Pit-1* has been associated with dwarfism in both humans and mice (Pfäffle et al., 1992). It has been reported that some mutations in the *Pit-1* gene cause the production of growth, prolactin, and thyroid-stimulating hormones (TSH) released by the pituitary to stop or underproduction (Renaville et al., 1997; Thuy et al., 2018). The *Pit-1* gene is thought to contribute to mammary gland development and milk production (Cohen et al., 1996). Because of these functions, the *Pit-1* gene may be considered as a candidate gene for increasing milk production and regulating growth and development in farm animals (Zhang et al., 2009; Heidari et al., 2012). *Pit-1* acts on PRL and GH, and since these hormones are also necessary for mammary gland development and milk production (Peel and Bauman, 1987; Pytlewski et al., 2018; Thuy et al., 2018), genetic variation of the *Pit-1* gene may be associated with yield traits and can be considered as a marker.

While it has been reported that *Pit-1* genotypes are effective on protein content and milk yield as well as some carcass characteristics in Holstein cattle (Renaville et al., 1997; Oprzadek et al., 2003; Bayram et al., 2017), some studies (Özdemir et al., 2018) have been reported to have no significant effects on milk yield traits. On the other hand studies on Simmental cattle found a significant relationship between *Pit-1* genotypes and examined milk yield traits (Cosier, 2006; Trakovicka et al., 2015), while other researchers reported no significant relationship (Vlaic et al., 2003). They observed insignificant effects of *Pit-1* gene polymorphism on milk yield and its composition in Brown Swiss cattle (Aytekin and Boztepe, 2013). In association studies on Angus, Limousine, and some other beef cattle, significant associations were reported between *Pit-1* polymorphic structures and birth weight and weaning weight (Dybus et al., 2003; Xue et al., 2006; Pytlewski et al., 2018). While other researchers reported *Pit-1* polymorphic structures, they stated that there was no significant relationship between the gene and meat yield characteristics (Di Stasio et al., 2002; Zhao et al., 2004; Curi et al., 2006).

This study aimed to examine the polymorphic structures of *Pit-1* and *CSN3* ( κ casein) genes of Holstein calves raised in organic conditions in Gümüşhane province, to determine the distribution of genotype and allele frequencies, and to investigate the effects of the determined polymorphic structures on calf birth weight.

## 2 Materials and methods

### 2.1 Material

This work was done in the Gümüşhane province, in Dogan Organic Products Inc. Individual blood samples and birth weight records of 100 Holstein calves born in the same year and season were used as material.

### 2.2 DNA isolation

Genomic DNA was obtained from blood samples obtained from Holstein calves by applying the QIAGEN-Gentra Puregene Kit.

### 2.3 Polymerase chain reaction (PCR) process

The primer sequences used in the PCR process were applied to amplify the relevant gene regions from genomic DNA (Table 1). The materials and amounts required for PCR are presented in Table 2, and the relevant PCR programs are presented in Table 3.

**Table 1:** Primer sequences of *Pit-1* and *CSN3* genes.

Primers		Size	Reference
*Pit-1*	F: 5 ${}^{\prime }$ -ACT CGC TAT TAC ACA ATA GGA GCC T-3 ${}^{\prime }$	260 bp	Özdemir (2012)
	R: 5 ${}^{\prime }$ -TCC TGC CAA CTC CTC ACC TCC C-3 ${}^{\prime }$		
*CSN3*	F: 5 ${}^{\prime }$ -ATT TAT GGC CAT TCC ACC AA-3 ${}^{\prime }$	351 bp	Doğru et al. (2008)
	R: 5 ${}^{\prime }$ -ATT AGC CCA TTT CGC CTT CT-3 ${}^{\prime }$		

**Table 2:** PCR components and their respective amounts for the *Pit-1* and *CSN3* gene regions.

Materials	Volume
dNTP	1 µ L
Taq	0.5–1 U
MgCl ${}_{2}$ (25 mM)	1 µ L
10× buffer	3 µ L
Primer F	1 µ L
Primer R	1 µ L
Genomic DNA	100–150 ng
Pure water	15 µ L

PCR processes for each gene region, 50–150 ng of each genomic DNA samples, were taken into separate tubes. The amount of material specified in Table 2 was added on it, and the tubes were centrifuged by flashing centrifugation. Afterwards, PCR processes were carried out for each sample with the PCR program specified in Table 3.

**Table 3:** PCR program for *Pit-1* and *CSN3* gene regions.

Gene regions	Temperature	Time	Cycle	Steps
*Pit-1*	94 ${}^{\circ }$ C	5 min	33 cycles	Initial denaturation
	94 ${}^{\circ }$ C	45 s		Denaturation
	60 ${}^{\circ }$ C	45 s		Annealing
	72 ${}^{\circ }$ C	50 s		Extension
	72 ${}^{\circ }$ C	5 min		Final extension
*CSN3*	94 ${}^{\circ }$ C	5 min	30 cycles	Initial denaturation
	94 ${}^{\circ }$ C	45 s		Denaturation
	60 ${}^{\circ }$ C	45 s		Annealing
	72 ${}^{\circ }$ C	60 s		Extension
	72 ${}^{\circ }$ C	7 min		Final extension

After completion of PCR amplification of each sample, 10 µ L of each PCR amplicon was electrophoresed using 1.2 % ethidium bromide stained agarose gel with a run condition of 80 V for 20 min, then visualized under UV light. Presence of expected band indicated positive PCR product.

### 2.4 PCR-RFLP process

The restriction enzyme used in the study to detect polymorphisms of both *Pit-1* and *CSN3* gene regions is *Hinf*I, and the 5 ${}^{\prime }$ –3 ${}^{\prime }$ recognition region is GATC sequence. For PCR-RFLP, approximately 8–10 µ L of each positive PCR amplicon was taken and placed in 0.2 mL sterile tubes, 6–8 U of *Hinf*I restriction enzyme, 6–8 µ L of RE buffer (Buffer Tango and Buffer R); 5–7 µ L of distilled water was added, and then the mixture was covered with 6–8 µ L of mineral oil. Then, incubation was carried out in an oven at 37 ${}^{\circ }$ C for 12 h.

To observe the crime after the cutting process of DNAs with *Hinf*I is completed, bromphenol dye, which is 3 µ L of loading buffer, was added to each of the samples that had undergone restriction cut, which was removed from the oven. All products were moved on the parafilm with the help of a micropipette to remove mineral oil. The gel was placed in the electrophoresis tank filled with 1XTBE buffer by loading the previously prepared 2 % agarose gel separately. It was then subjected to electrophoresis at 45 V for 90 min. After the electrophoresis, gel was taken and each product was genotyped with the help of a standard marker under UV light (Özdemir, 2012).

### 2.5 Statistical analysis

Allelic and genotype frequencies and a Hardy–Weinberg equilibrium exact test were estimated using the software GenPop V4.3 (Raymond and Rousset, 1995). In the analysis of association with yield, the obtained data were subjected to analysis of variance (ANOVA), and for this purpose SPSS statistical package (IBM SPSS Statistics for Windows, Version 25.0; Armonk, NY; IBM Corp.) program was used. Birth weight characteristics were taken as the basis of the yield characteristics of the calves, and genotype differences were aimed to be revealed.

The following statistical model was used according to the yield characteristics in the study:

$$Y_{ij}=\mu +a_{i}+e_{ij},$$

where $Y_{ijk}$ is the observed value of phenotype for any calf, μ is population mean, $a_{i}$ is genotype effect ( i = AA, AB, BB), and $e_{ij}$ is the random residual effect.

## 3 Results and discussion

### 3.1 PCR results

**Figure 1:**
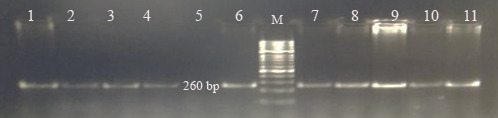
Agarose gel appearance of *Pit-1*-PCR products under UV (M: marker, *Pit-1*: 260 bp).

**Figure 2:**
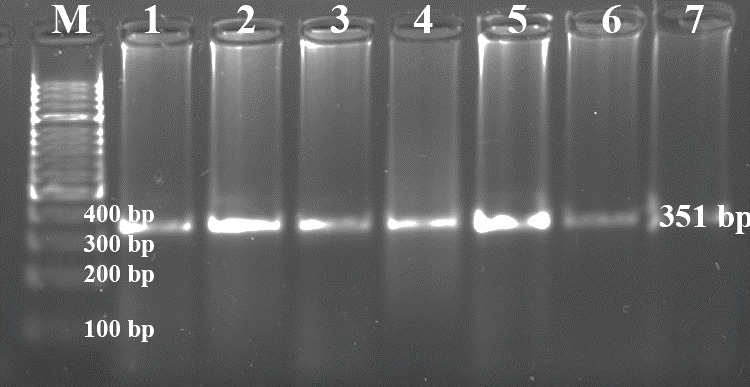
Agarose gel appearance of *CSN3*-PCR products under UV (M: marker, *CSN3*: 351 bp).

Each of the genomic DNA samples obtained from Holstein calf blood was performed separately for the *Pit-1* and *CSN3* gene regions, and DNA bands were obtained by performing PCR on 1.2 % agarose gel. Figures 1 and 2 show the agarose gel image of the PCR products under UV light.

### 3.2 PCR-RFLP results

**Figure 3:**
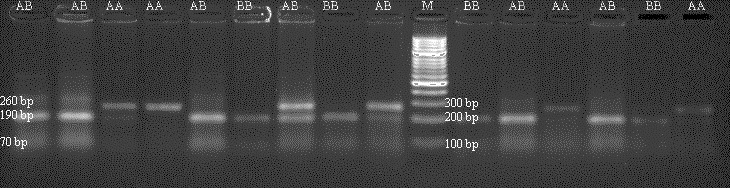
PCR-RFLP gel image of *Pit-1*/*Hinf*I polymorphism. AA: 260 bp, AB: 160/190/70 bp, BB: 190/70 bp.

DNA samples obtained from Holstein calves were amplified separately for *Pit-1* and *CSN3* genes in a PCR device, and polymorphic regions of *Pit-1* and *CSN3* genes were determined by digesting with *Hinf*I restriction endonuclease enzyme. Theoretically, with the *Pit-1*/*Hinf*I polymorphism, the AA genotype is characterized by 260 bp, BB genotype 190/70 bp, and AB genotype 260/190/70 bp bands. In Fig. 3, an exemplary agarose gel image of the *Pit-1*/*Hinf*I polymorphism PCR-RFLP result under UV light is presented.

**Figure 4:**
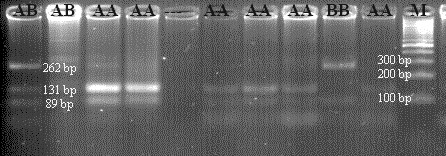
PCR-RFLP gel image of *CSN3*/*Hinf*I polymorphism. AA: 131/89 bp, AB: 262/131/89 bp, BB: 262/89 bp.

For *CSN3*, theoretically yield tapes have bp lengths of 262/89 for BB, 131/89 for AA, and 262/131/89 for AB. The cut-off site in 131 bp was defined as polymorphic, while the cut-off region in 262 bp was seen as the standard cut-off site. An exemplary agarose gel image of the PCR-RFLP result of the *CSN3*/*Hinf*I polymorphism is presented in Fig. 4.

### 3.3 Gene and genotype frequencies and genetic equilibrium test results

In the study, three different genotypes – AA, AB, and BB – were identified on both *Pit-1*/*Hinf*I and *CSN3*/*Hinf*I gene regions. Detected genotypes and allele gene frequencies are presented in Table 4; Hardy–Weinberg genetic equilibrium test and $X^{2}$ test results are presented in Table 5.

**Table 4:** *Pit-1* and *CSN3* genes, genotype, and allele gene frequencies of calves.

Genotype	*Pit-1*		*CSN3*	
	n	%	n	%
AA	11	13.4	24	24.5
AB	33	40.2	36	36.7
BB	38	46.3	38	38.8
Allele gene	A	B	A	B
Frequencies (%)	34	66	43	57

When the calf population was analyzed in terms of *Pit-1*/*Hinf*I polymorphism allele gene frequencies, it was determined that the A allele was 0.34 and the B allele was 0.66 (Table 4). While it was observed that the B allele was observed at a high frequency in the race, in general, the AA genotype was 13.4 %, the AB genotype was 40.2 %, and the BB genotype was 46.3 %. When the *CSN3*/*Hinf*I polymorphism was examined in terms of allele gene frequencies, it was determined that the A allele had a frequency of 0.43 and the B allele had a frequency of 0.57 (Table 4). While it was observed that the B allele was observed at high frequency in the herd, in general, 24.5 % of the AA genotype, 36.7 % of the AB genotype, and 38.8 % of the BB genotype were detected. When the allele gene frequencies and genotype frequencies of both polymorphic regions are evaluated, it can be said that the rate of heterozygous individuals in the herd is high in terms of both *Pit-1*/*Hinf*I polymorphism and *CSN3*/*Hinf*I polymorphism, and the herd has sufficient biological diversity for breeding programs.

**Table 5:** Hardy–Weinberg genetic equilibrium test results of gene regions.

Genes	n	Observed			Expected			$X^{2}$ test	
		AA	AB	BB	AA	AB	BB	value	
*Pit-1*	82	11	33	38	9.2	36.3	36.2	0.78	NS
*CSN3*	98	24	36	38	18.0	48.0	32.0	6.13	∗

NS: non-significant ( P>0.05 ), ${}^{*}$ P<0.05 .

Concerning *Pit-1* gene polymorphism, previously in Holstein cattle (Renaville et al., 1997; Jia et al., 2011; Özdemir, 2012; Bayram et al., 2017; Özdemir et al., 2018), in Brown Swiss cattle (Aytekin and Boztepe 2013), in Black and White cattle (Zwierzchowski et al., 2002; Dybus et al., 2004), and in Simmental cattle (Viorica, 2006; Vlaic et al., 2007; Trakovicka et al., 2015), it has been reported that *Pit-1* gene was detected in three genotypes, and BB genotype and B allele gene frequency are higher. Except in Bayram et al. (2017), the results obtained from these studies were found to be in agreement with our study result.

In the studies of *CSN3*/*Hinf*I polymorphism in Brown Swiss cattle (Özdemir and Doğru, 2005; Akyüz et al., 2013), in Holstein cattle (Gürcan, 2001; Özdemir and Doğru, 2005; Gedik, 2009; Akyüz et al., 2013; Demirel, 2019; Ünal and Kopuzlu, 2022), and in Simmental cattle (Akyüz et al., 2013; Akyüz and Çınar, 2014), it has been reported that *CSN3* gene has AA, AB, and BB genotypes, and B allele gene frequency is found at a higher frequency in most studies. When the related studies are examined, it is seen that the findings of the gene and genotype frequency are compatible with our study results.

It was determined that the distribution of genotype frequencies of the *Pit-1*/*Hinf*I polymorphism of Holstein calves was in equilibrium ( P>0.05 ) according to the Hardy–Weinberg genetic equilibrium test, but the distribution of genotype frequencies of the *CSN3*/*Hinf*I polymorphism was not in equilibrium ( P<0.05 ) (Table 5). This may be due to a breeding program being implemented in the herd or a sampling error.

### 3.4 The effect of *Pit-1*/*Hinf*I and *CSN3*/*Hinf*I gene phenotypes on calf birth weight

The relationships between the genotypes of the *Pit-1*/Hinf1 and *CSN3*/Hinf1 polymorphisms and calf birth weight were examined with the variance analysis results. The least squares means and standard errors of calf birth weight of *Pit-1*/*Hinf*I and *CSN3*/*Hinf*I genotypes are presented in Table 6.

**Table 6:** The least squares means and standard errors of the *Pit-1* and *CSN3* genotypes in terms of calf birth weight (kg).

Genotype	*Pit-1*				*CSN3*			
	n	$\bar{x}$	±	$S\bar{x}$	n	$\bar{x}$	±	$S\bar{x}$
AA	11	41.45		0.813	24	42.29		0.738
AB	33	41.09		0.375	36	41.06		0.376
BB	38	42.26		0.563	38	41.95		0.399
General	82	41.68		0.323	98	41.70		0.277

The overall mean birth weight of *Pit-1*/*Hinf*I polymorphism in Holstein calves was determined as 41.68 ± 0.323 kg. According to the data obtained, the highest mean BB genotype (42.26 ± 0.563 kg) and the lowest mean AB genotype (41.09 ± 0.375 kg) were determined among the *Pit-1* genotypes in terms of birth weight. The mean birth weight of calves with BB genotype was found to be higher than that of calves with AA and AB genotypes (Table 6), but these differences were not found to be statistically significant ( P>0.05 ). Xue et al. (2006) reported that in Nanyang cattle, BB genotypes of *Pit-1*/*Hinf*I polymorphism had higher calf birth weight averages than AA genotype calves ( P<0.05 ). The fact that the birth weight averages of the BB genotypes were found to be high in both studies indicates that the B allele positively affects the growth characteristics of cattle. However, Pytlewski et al. (2018), reported that AA homozygotes of the *Pit-1* gene are characterized by the biggest calf weight. A cow's body weight is an important factor that can affect her milk and reproductive production. They tried to prove the existence of associations between *Pit-1* gene polymorphism, reproductive potential, and body weight of cows and calves and observed more favorable results in *Pit-1* AA homozygotes. They also suggested that it is possible to use these associations in the genetic selection of farm animals.

The general mean of birth weight of *CSN3*/*Hinf*I polymorphism was determined as 41.70 ± 0.277 kg. According to the data obtained, the highest mean AA genotype (42.29 ± 0.738 kg) and the lowest mean AB genotype (41.06 ± 0.376 kg) were determined among the *CSN3*/*Hinf*I genotypes in terms of birth weight. The mean birth weight of the calves with AB genotype was 1230 and 890 g less than the calves with AA and BB genotypes, respectively (Table 6), but these differences were not found to be statistically significant ( P>0.05 ). When similar studies on this subject were examined before, no different study was found that examined the relationship between *CSN3*/*Hinf*I polymorphism and calf birth weight. It has been suggested that the *CSN3* gene polymorphism as a molecular marker can provide significant advances in improvement of milk yield traits in cattle (Lunden et al., 1997; Strzalkowska et al., 2002; Özdemir and Doğru, 2005; Caroli et al., 2009). However, a growing number of selection programs for increasing milk production did not pay attention to the fertility of dairy animals. Selection for improving milk yield may be causing a general loss of reproductive fitness (Nasr et al., 2016).

## 4 Conclusion

Genotypes of *Pit-1*/*Hinf*I polymorphism (AA, AB, and BB genotypes) and *CSN3*/*Hinf*I polymorphism (AA, AB, and BB genotypes) were determined on individual blood samples of Holstein calves using the PCR-RFLP method. The genotype and allele gene frequencies of the *Pit-1*/*Hinf*I and *CSN3*/*Hinf*I polymorphisms revealed the genotype diversity of the breed. No statistically significant correlation was found between the genotypes of *Pit-1*/*Hinf*I and *CSN3*/*Hinf*I polymorphisms detected in Holstein calves and calf birth weight. It is suggested that these and similar polymorphic structures can be used in animal breeding by associating them with different performance characteristics on different breeds in different regions.

## Data Availability

The data sets are available upon request from the corresponding author.
